# Regulation of Transient Site-specific Copy Gain by MicroRNA*^♦^

**DOI:** 10.1074/jbc.M115.711648

**Published:** 2016-01-11

**Authors:** Joshua C. Black, Hailei Zhang, Jaegil Kim, Gad Getz, Johnathan R. Whetstine

**Affiliations:** From the Massachusetts General Hospital Cancer Center and Departments of ‡ Medicine and; ¶ Pathology, Harvard Medical School, Massachusetts 02129 and; the § Broad Institute of MIT and Harvard, Cambridge, Massachusetts 02142

**Keywords:** chromatin, drug resistance, genomic instability, histone demethylase, microRNA (miRNA), CKS1B, JMJD2A, KDM4A, TSSG, cisplatin

## Abstract

Intra-tumor copy number heterogeneity is commonly observed in cancer; however, the molecular mechanisms that contribute to heterogeneity remain poorly understood. Up-regulation of the histone demethylase KDM4A promotes transient site-specific copy gain (TSSG) in cells; therefore, uncovering how KDM4A levels are controlled is important for understanding the regulation of copy number heterogeneity. Here, we demonstrate that KDM4A is regulated by hsa-mir-23a-3p, hsa-mir-23b-3p, and hsa-mir-137. Altering expression of these microRNAs (miRNAs) regulates KDM4A-dependent TSSG. miRNA inhibition promoted copy gains and increased expression of the drug-resistant oncogene *CKS1B*, which was further substantiated in primary breast tumors. Consistent with increased *CKS1B* expression, miRNA inhibition reduced breast cancer cell sensitivity to cisplatin. Our data identify these miRNAs as regulators of TSSG and copy gains of a drug resistance gene.

## Introduction

Genomic instability is a hallmark of cancer and contributes to drug resistance (1). Both adult and pediatric cancers have recurrent gains and losses of chromosomal regions, but little is known regarding the molecular mechanisms causing either transient or permanent copy number changes at specific sites within the genome. Such copy number gains, when contributing to increased expression of oncogenes, have been shown to impact cellular behavior and/or correlate with poor outcome and reduced chemotherapeutic response (2–5). For instance, tumors with worse outcome and reduced response to therapeutics often harbor chromosome 1q12-25 cytogenetic gains; however, the genes that contribute to this phenotype may vary depending on tumor type even though the same cytogenetic region is gained (2–10).

We previously discovered that overexpression of the lysine demethylase KDM4A/JMJD2A and the modulation of epigenetic states (*i.e.* histone 3 lysine 9 and 36 methylation) results in transient site-specific copy gains (TSSGs)^5^ through rereplication in the human genome (11–14). TSSGs are copy gain/amplification events that are reversible, occur during the cell cycle, but are not permanently integrated into the genome (11–14). Overexpression of histone lysine demethylase KDM4A was sufficient to promote rereplication and copy gain of specific chromosomal regions that are implicated in drug resistance and worse clinical outcome (*e.g.* 1q12h and 1q21) (2–5, 11–13). The *KDM4A* locus (1p34.1) is amplified in human tumors (∼20%) and significantly correlates with copy gains that were recapitulated as TSSGs in transgenic cell lines (12). Moreover, TSSG is not just a cancer-specific event but can be regulated by physiologic stimuli. For example, hypoxia also promotes TSSGs through stabilization of KDM4A protein levels (11, 13). Thus, understanding how KDM4A is regulated will help understand TSSGs, intra-tumor copy number heterogeneity, and provide insights into the amplification of potential drug resistance genes, especially in the 1q12-21 locus.

KDM4A protein levels are regulated, during cell cycle and in hypoxic exposure, by the SKP1-Cul1-F-box ubiquitin ligase complex and at least three F-box proteins (11, 15–18). However, it is likely that other mechanisms exist to modulate KDM4A protein levels, which will play an important role in regulating TSSG in 1q12-21. Possible candidates for regulating KDM4A are microRNAs (miRNAs). MicroRNAs are short (19–22 nucleotides) non-coding RNAs, which in complex with the RNA-induced silencing complex target the 3′-untranslated region (3′-UTR) through binding to specific complementary seed sequences (19). Transcripts targeted by RNA-induced silencing complex/miRNA are then translationally repressed or degraded (19).

Here, we demonstrate that KDM4A is regulated by hsa-mir-23a/b-3p (hereafter hsa-mir-23a/b) and hsa-mir-137. Addition of miRNA mimics to cells resulted in decreased KDM4A protein expression, whereas inhibition of the endogenous miRNA resulted in increased KDM4A protein levels. Addition of the KDM4A 3′-UTR to luciferase rendered it responsive to these miRNAs, which was blocked by mutation of the hsa-mir-23a/b and hsa-mir-137 seed sequences. Interestingly, up-regulation of KDM4A through depletion of these miRNAs promotes TSSG of 1q12-21. Reciprocally, treatment with hsa-mir-23a/b or hsa-mir-137 mimics was sufficient to abrogate KDM4A-dependent TSSGs in response to hypoxia. Consistent with these observations, we used miRNA inhibitors in MDA-MB-231 breast cancer cells to promote gain of 1q12-21 as well as the amplification and increased expression of *CKS1B*, which is a drug-resistant oncogene (4, 20–23). Furthermore, analysis of primary breast tumors (BRCA) in The Cancer Genome Atlas (TCGA) revealed that deletion of hsa-mir-23a correlates with increased copy number of 1q12-21 in primary tumors and associates with copy gain and increased expression of the drug- resistant oncogene *CKS1B*. Consistent with these observations, miRNA inhibitors reduced breast cancer cell response to cisplatin. Our results implicate miRNA regulation as a modulator of TSSGs and suggest that miRNA therapy could be used to reduce KDM4A-driven copy number heterogeneity and potentially affect drug resistance.

## Experimental Procedures

### 

#### 

##### Cell Culture and Transfections

hTERT-RPE-1 (called RPE throughout) and MDA-MB-231 cells were maintained in DMEM with 10% fetal bovine serum, 1% penicillin/streptomycin, and l-glutamine. SK-N-AS cells were maintained in DMEM/F-12 (Gibco) with 10% fetal bovine serum, 1% penicillin/streptomycin, and l-glutamine. H2591 cells were maintained in RPMI 1640 medium (Gibco) with 10% fetal bovine serum, 1% penicillin/streptomycin, and l-glutamine. Transient transfection experiments with miRNA mimics or inhibitors were performed using X-tremeGENE siRNA reagent (Roche Applied Science) in Opti-MEM I media overnight (∼12 h). Media were changed to DMEM or DMEM/F-12 or RPMI 1640 medium as appropriate following the overnight incubation, and cells were collected at 72 h following transfection. Each miRNA experiment represents the average of at least two different transfections for each miRNA mimic or inhibitor. Transient transfection experiments with KDM4A siRNA were co-transfected with the miRNA using X-tremeGENE siRNA reagent (Roche Applied Science) in Opti-MEM I media overnight. Silencer Select siRNA for KDM4A was purchased from Life Technologies, Inc. (s18636).

##### Hypoxic Conditions

Cells were plated onto culture dishes and allowed to adhere for 20–24 h in normoxia (5% CO_2_, 21% O_2_, and 74% N_2_). For hypoxic treatment, cells were maintained in a HERA Cell 150 incubator (Thermo Scientific) flushed with 5% CO_2_, 1% O_2_, and balanced with N_2_ for the duration of the experiment. Incubator calibrations and verifications were carried out by Bianchi Associates calibrations/verifications.

##### Fluorescent In Situ Hybridization (FISH)

FISH was performed as described in Ref. 12. Probes for 1q12h, chromosome 8 centromere (α satellite), and *CKS1B* were purchased from Rainbow Scientific through Oxford Gene Technologies. Probes for 1q21.2 (*BCL9*) and 1q23.3 were purchased from Agilent (SureFISH). Images of multiple planes of fields of nuclei were acquired on an Olympus IX81 spinning disk microscope using a ×40 objective and analyzed using Slidebook 5.0 software. We used a conservative scoring metric for copy gain. Any foci that were touching were scored as a single copy to prevent increased numbers due to normally replicated foci. For RPE cells, copy gain was scored as any cell with three or more distinct foci. For MDA-MB-231 cells, copy gain was scored for any cell with seven or more foci for 1q12h and *CKS1B* and five or more for 8c or *CDKN2C*. For SK-N-AS cells, copy gain was scored for any cell with four or more foci for 1q12h and three or more for 8c. For H2591 cells, copy gain was scored for any cell with five or more foci for 1q12h and four or more for 8c. Approximately 100 cells for each replicate were scored for all experiments. All FISH experiments include at least two biological replicates.

##### Antibodies

Antibodies used were as follows: KDM4A (Neuro mAb, 75-189), β-actin (Millipore), actinin (Santa Cruz Biotechnology, sc-17829), and CAIX (Abcam ab108351).

##### Western Blots

Western blots were performed as in Ref. 15. Samples for Western blotting analysis were from the same collections used for FISH, FACS, and RNA analyses. Quantitation was performed using ImageJ gel analysis with area under the curve. KDM4A levels were normalized to actin or actinin levels (as indicated in each figure), and then a ratio to the appropriate control sample was calculated. Data are thus presented as a fold change relative to the control.

##### Expression Plasmids and Luciferase Assays

The WT and MT KDM4A 3′-UTR were cloned into pMIR as the 3′-UTR to luciferase. The pMIR-3′-UTR constructs and a β-galactosidase construct for normalization were co-transfected with the indicated miRNA mimics for 48 h using X-tremeGENE siRNA transfection reagent (Roche Applied Science) in Opti-MEM I media (Life Technologies, Inc.). Cells were collected by scraping, and lysates were prepared following the Dual-Light system instructions (Life Technologies, Inc.). The Dual-Luciferase and β-galactosidase assays were performed using the Dual-Light system following the manufacturer's instructions (Life Technologies, Inc.). Measurements for two biological replicates were taken in triplicate and averaged.

##### miRNA Mimics and Inhibitors

The miRNA mimics and inhibitors were purchased from Life Technologies, Inc. The mimics used were MirVana pre-miRNA23a (MC10644), MirVana pre-miRNA23b (MC10711), MirVana pre-miRNA137 (MC10513), MirVana pre-miRNA200b (MC10492), and MirVana pre-miRNA200c (MC11714) and MirVana Control (4464058). The inhibitors used were MiRVana anti-miRNA23a (MH10644), MiRVana anti-miRNA23b (MH10711), MiRVana anti-miRNA137 (MH10513), MiRVana anti-miRNA200b (MH10492), MiRVana anti-miRNA200c (MH11714), and MirVana Control (4464076).

##### Cisplatin Sensitivity by MTT Assay

5000 MDA-MB-231 cells were plated overnight in each well of a 96-well plate. Cells were transfected with miRNA inhibitors using X-tremeGENE siRNA reagent (Roche Applied Science) in Opti-MEM I media overnight (∼12 h). Media were changed to DMEM following the overnight incubation, and cells were allowed to recover for 8 h. Cisplatin (Abcam ab141398) was resuspended in 0.9% NaCl right before use. Cisplatin was added following the 8-h recovery to a final concentration of 300 μm. Cells were processed using Cell Proliferation Kit I MTT (Roche Applied Science) 48 h after addition of cisplatin following the manufacturer's instructions. Each experiment consisted of four technical replicate wells that were averaged together and then taken as a ratio to the no-cisplatin sample. The data presented are the average of eight biological replicates.

##### RNA Extraction and Quantitative PCR

RNA extraction, cDNA synthesis, and quantitative PCR were conducted as in Ref. 11. Expression levels were analyzed by quantitative real time PCR in a LightCycler 480 with FastStart Universal SYBR Green Master (Roche Applied Science) following the manufacturer's protocols. All samples were normalized by comparison with β-actin transcript levels. Primers are available upon request.

##### TCGA Data Set and Copy Number Determination

The copy number and mRNA expression for TCGA breast cancer (BRCA) were download from Broad GDAC (Genome Data Analysis Center) Firehose analysis run of 2014_07_15 (doi:10.7908/C1TQ60P0). 1,030 common samples from two data platforms were used in this analysis. The somatic copy number alterations for 23,246 genes and 928 microRNAs were annotated by GISTIC2.0 (24–26). The copy number change in each gene/miRNA is defined as possessing deep deletion (−2), shallow deletions (−1), neutral copy number (0), low gain (+1), and high gain (+2) in each sample using sample-specific thresholds. High gains are segments with a copy number that exceed the maximum median chromosomal arm copy number for that sample by at least 0.1; low gains are segments with copy numbers from 2.1 to the high gain threshold; neutral segments have copy numbers between 1.9 and 2.1; shallow losses have copy numbers between 1.9 and the deep deletion threshold; and deep deletions have copy numbers that are below the minimum median chromosomal arm copy number for that sample by at least 0.1.

##### Determination of Cytoband Copy Number and Correlation with MicroRNA Loss

In addition to the copy number annotation for each gene, the mean focal copy number for 807 cytobands, including the X chromosome, were annotated in each sample by taking an average of focal copy numbers of every gene within the same cytoband. Arm-level somatic copy number alteration contributions to the mean focal copy number in each cytoband were removed by only considering GISTIC annotated focal copy numbers that are smaller than a chromosome arm or entire chromosome. Detecting chromosomal regions significantly co-amplified with microRNA copy loss or deletion was performed by approximating a null distribution of mean cytoband copy differences by a normal function as shown in Equation 1,

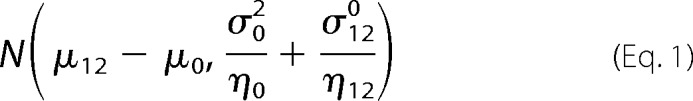
 where μ_0_ and μ_12_ are sample means across all cytobands; σ_0_^2^ and σ_0_^12^ are mean sample-specific variances with each group, and η_0_ and η_12_ are the number of samples in microRNA copy-neutral (GISTIC annotation = 0) and microRNA copy-loss (GISTIC annotation = −1 or −2) groups, respectively. This test is based on comparing the means of the two sets while permuting values within each of the samples (and using a Gaussian approximation). The *p* values across 807 cytobands were annotated by computing the probability of more extreme differences than the corresponding cytoband copy difference in the null distribution. The *QQ* plot of those *p* values was used to show that many genes follow the null hypothesis, and their associated *p* values behave appropriately.

## Results

### 

#### 

##### KDM4A Is Regulated by miRNAs

**FIGURE 1.**
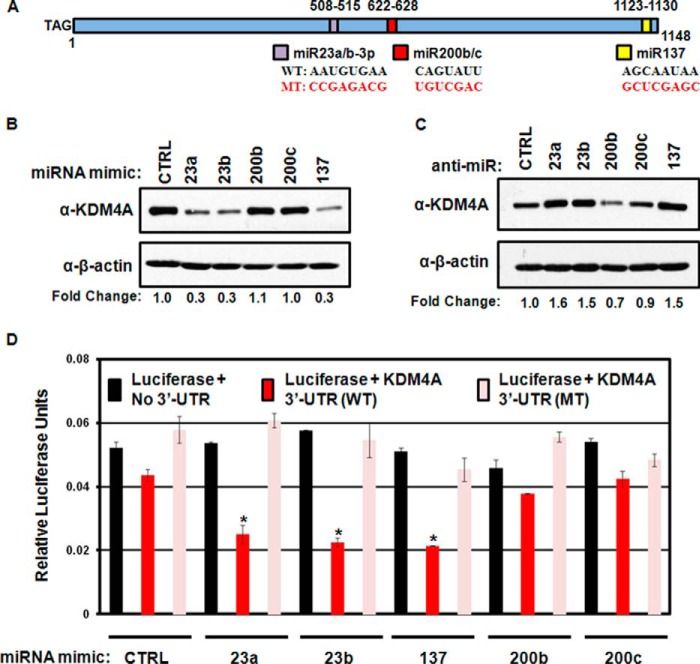
**Regulation of KDM4A by miRNA.** *A*, schematic of KDM4A 3′UTR. The nucleotide length and the positions of TARGETSCAN 6.2 predicted seed sequences are indicated. The seed sequences are indicated as are the mutations performed to generate the mutant 3′-UTR (*MT*) in the schematic. *B*, Western blotting analysis of KDM4A protein levels following treatment with the indicated miRNA mimics. Representative Western blots are from one of two biological replicates. *C*, Western blotting analysis of KDM4A protein levels following treatment with the indicated miRNA. *D*, luciferase analysis of KDM4A WT and KDM4A mutant (*MT*) 3′-UTR response to miRNA mimics. Data were normalized to the co-transfected β-galactosidase levels for relative light units. Data represent average of two biological replicates assayed in technical triplicates. *Error bars* represent the S.E. * indicates significant difference from control (*CTRL*) by two-tailed Student's *t* test (*p* < 0.05).

KDM4A is an important regulator of TSSGs (11–14). Uncovering how KDM4A protein levels are regulated is crucial to understanding how TSSGs can be regulated. KDM4A levels are largely regulated post-transcriptionally (11, 12, 15–18), suggesting that miRNAs may be ideal candidates to contribute to this regulation. To address this hypothesis, we analyzed the TARGETSCAN database for miRNAs that could target KDM4A. TARGETSCAN6.2 identified three conserved miRNA seed sequences in the *KDM4A* 3′-UTR (Fig. 1*A*) for hsa-mir-23a/b-3p (hereafter hsa-mir-23a/b), hsa-mir-137, and hsa-mir200b/c (27, 28). To determine whether these miRNAs could indeed regulate KDM4A, we treated the immortalized but non-transformed human retinal pigment epithelial cell line (RPE cells) with miRNA mimics (Fig. 1*B*). KDM4A protein levels were down-regulated when cells were exposed to hsa-mir-23a/b and hsa-mir-137 but had minimal change when exposed to increased hsa-mir200b/c. Moreover, KDM4A protein levels increased when cells were treated with inhibitors of hsa-mir-23a/b and hsa-mir-137 but not with hsa-mir200b/c (Fig. 1*C*). These results are consistent with hsa-mir-23a/b and hsa-mir-137 regulating KDM4A in human cells.

To determine whether the *KDM4A* 3′-UTR was the direct target of hsa-mir-23a/b and hsa-mir-137, we cloned the *KDM4A* 3′-UTR downstream of the luciferase cDNA. The miRNA seed sequences were left intact (WT UTR) or carried a series of point mutations removing the seed sequences for hsa-mir-23a/b, hsa-mir-137, and hsa-mir 200b/c (MT UTR; Fig. 1*A*). These constructs were then introduced into RPE cells in conjunction with mimics to hsa-mir-23a/b, hsa-mir-137, hsa-mir200b/c, or a control miRNA mimic. Cells treated with hsa-mir-23a/b or hsa-mir-137 miRNAs reduced luciferase expressions when luciferase was fused with the WT 3′-UTR but not when attached to the mutated 3′-UTR (*MT*; Fig. 1*D*). Overexpression of hsa-mir200b/c did not induce significant change in luciferase expression. Taken together, our data demonstrate that KDM4A is a direct target for regulation by hsa-mir-23a/b and hsa-mir-137 in RPE cells.

##### Regulation of TSSGs by miRNA

**FIGURE 2.**
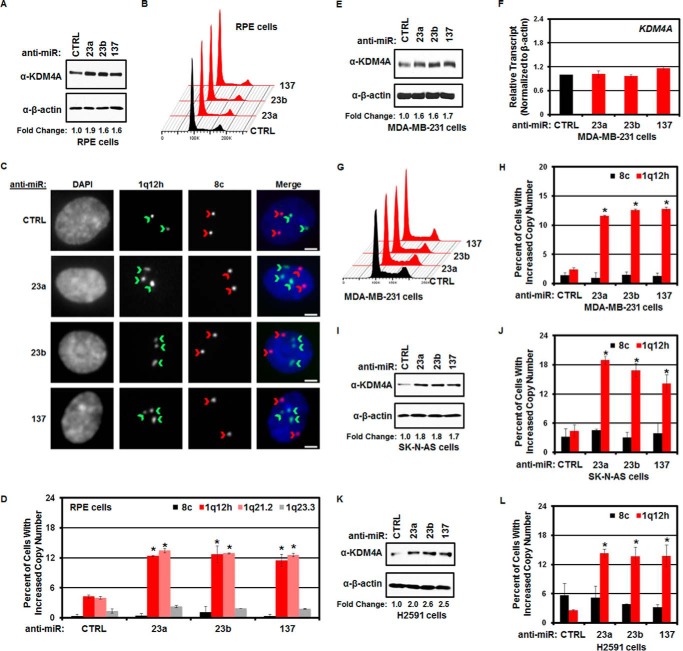
**Regulation of KDM4A by miRNAs promotes copy gain.** *A*, Western blotting analysis of KDM4A levels in response to miRNA inhibitors in RPE cells. Representative Western blots are from one of two biological replicates. *B*, treatment of RPE cells with the indicated anti-miRs does not affect cell cycle distribution. Representative cell cycle distribution was from one of two biological replicates. *C*, representative images of FISH for 1q12h and 8c in anti-miR-treated RPE cells. Note the increased number of 1q12h foci (indicated by *green chevrons*) but not chromosome 8c foci (indicated by *red chevrons*) in the anti-miR treated cells. *Scale bars* represent 5 μm. *D*, treatment of RPE cells with anti-miRs induces copy gain of 1q12-21. Quantification of FISH analysis. Data represent the average of two biological replicates. *Error bar*s represent the S.E. * indicates significant difference from control (*CTRL*) by two-tailed Student's *t* test (*p* < 0.05). *E*, Western blotting analysis of KDM4A levels in response to miRNA inhibitors in MDA-MB-231 cells. Representative Western blots are from one of two biological replicates. *F*, steady-state *KDM4A* transcript levels do not change in response to miRNA inhibitors in MDA-MB-231 cells. Data represent the average of two biological replicates. *Error bars* represent the S.E. * indicates significant difference from control (*CTRL*) by two-tailed Student's *t* test (*p* < 0.05). *G*, cell cycle distribution of MDA-MB-231 cells treated with anti-miRs. Representative distribution was from one of two biological replicates. *H*, treatment of MDA-MB-231 cells with anti-miRs induces copy gain of 1q12h. Quantification of FISH analysis is shown. Data represent the average of two biological replicates. *Error bars* represent the S.E. * indicates significant difference from control by two-tailed Student's *t* test (*p* < 0.05). *I*, Western blotting analysis of KDM4A levels in response to miRNA inhibitors in SK-N-AS neuroblastoma cells. Representative Western blots are from one of two biological replicates. *J*, treatment of SK-N-AS cells with anti-miRs induces copy gain of 1q12h. Quantification of FISH analysis. Data represent the average of two biological replicates. *Error bars* represent the S.E. * indicates significant difference from control by two-tailed Student's *t* test (*p* < 0.05). *K*, Western blotting analysis of KDM4A levels in response to miRNA inhibitors in H2591 lung cancer cells. Representative Western blots are from one of two biological replicates. *L*, treatment of H2591 cells with anti-miRs induces copy gain of 1q12h. Quantification of FISH analysis. Data represent the average of two biological replicates. *Error bars* represent the S.E. * indicates significant difference from control by two-tailed Student's *t* test (*p* < 0.05).

We previously demonstrated that increased expression of KDM4A was sufficient to promote TSSGs (11, 12). TSSGs are characterized by cells with at least one additional copy of specific genomic loci that occur during S phase (11–14). The ability of miRNAs to regulate KDM4A protein levels suggested that decreasing hsa-mir-23a/b or hsa-mir-137 expression would be sufficient to increase KDM4A levels and thus promote TSSGs. Therefore, we introduced hsa-mir-23a/b or hsa-mir-137 inhibitors (anti-miRs) into RPE cells for 72 h and assessed copy number by fluorescent *in situ* hybridization (DNA-FISH). The anti-miRs were sufficient to induce increased expression of KDM4A (Fig. 2*A*) without altering cell cycle distribution (Fig. 2*B*). We then determined the percentage of cells in the population that had at least one additional copy of the regions known to undergo TSSGs (*i.e.* 1q12h and 1q21.2) as well as control regions (*i.e.* 1q23.3 and chromosome 8 centromere) by DNA-FISH. Inhibition of hsa-mir-23a/b or hsa-mir-137 was sufficient to promote copy gain of 1q12h and 1q21.2 but did not alter the copy number of 1q23.3 or chromosome 8 centromere (Fig. 2, *C* and *D*). We confirmed these findings in MDA-MB-231 breast cancer cells. As in RPE cells, introduction of the anti-miRs resulted in increased KDM4A protein levels (Fig. 2*E*) without altering steady-state *KDM4A* transcript levels or cell cycle (Fig. 2, *F* and *G*). MicroRNA inhibition increased copy number of 1q12h (Fig. 2*H*) but not the chromosome 8 centromere. Treatment with microRNA inhibitors in neuroblastoma (SK-N-AS cells; Fig. 2, *I* and *J*) and lung cancer cells (H2591; Fig. 2, *K* and *L*) also increased KDM4A protein levels and promoted copy gain of 1q12h but not chromosome 8 centromere.

**FIGURE 3.**
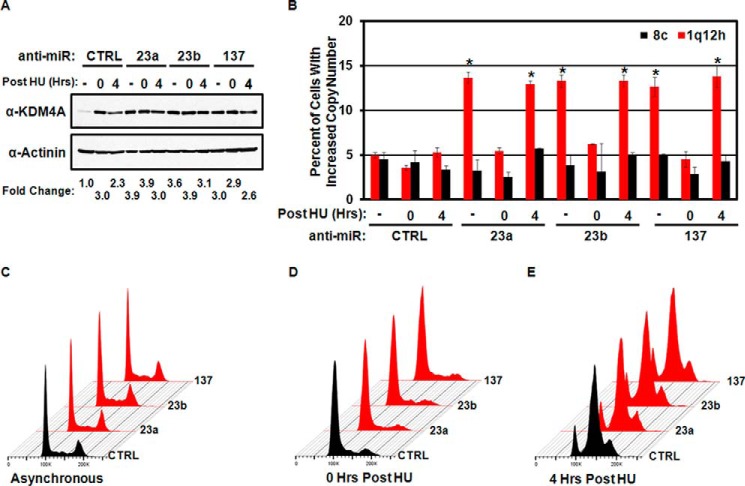
**MicroRNA-dependent regulation of KDM4A promotes TSSG.** *A*, Western blot depicting KDM4A levels in asynchronous or HU arrested and released cells treated with miRNA inhibitors. Representative Western blots are from one of two biological replicates. *B*, copy gain induced by miRNA inhibitors is transient. Quantification of FISH analysis from asynchronous RPE cells or HU-arrested (*HU 0*) or HU released for 4 h (*HU 4*). Data represent the average of two biological replicates. *Error bars* represent the S.E. * indicates significant difference from untreated control (*CTRL*) by two-tailed Student's *t* test (*p* < 0.05). *C–E*, treatment of RPE cells with the indicated anti-miRs does not affect cell cycle distribution (*C*) or HU arrest (*D*) or HU release (*E*). Representative cell cycle profiles are from one of two biological replicates.

TSSGs are characterized by their transient appearance during S phase (11–14). Therefore, we tested whether the observed copy gains were S phase-dependent and by definition TSSGs. RPE cells were transfected with hsa-mir-23a/b or hsa-mir-137 anti-miRs prior to arrest with hydroxyurea (HU) for 20 h or arrested and released from HU for 4 h. TSSG at 1q12h was assessed by DNA-FISH (Fig. 3, *A–E*). Early S arrest with HU blocked the ability of the miRNA inhibitors to induce copy gain (Fig. 3*B*). However, once cells were released into S phase, hsa-mir-23a/b or hsa-mir-137 anti-miRs promoted copy gain (Fig. 3*B*). These results demonstrate that inhibition of miRNAs promotes TSSG.

**FIGURE 4.**
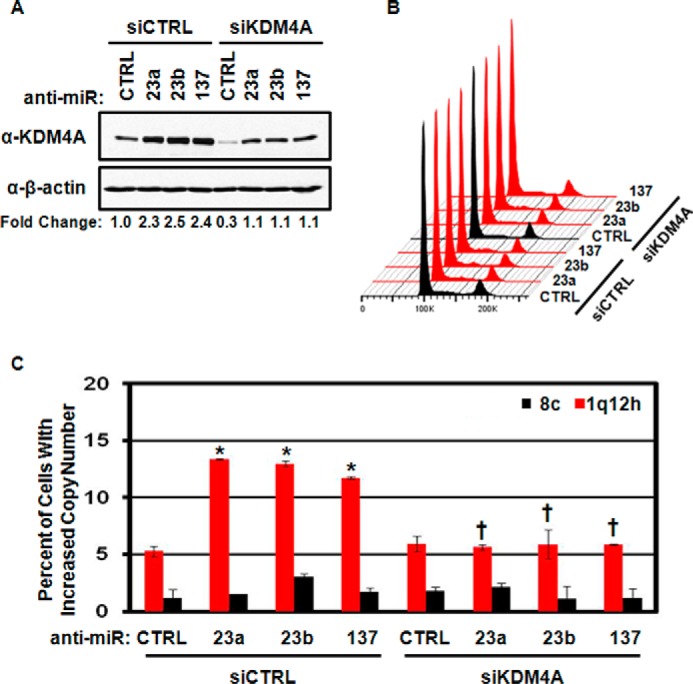
**Regulation of TSSG by miRNA is KDM4A-dependent.** *A*, Western blots depicting KDM4A levels from combined anti-miR and KDM4A depletion. Representative Western blots are from one of two biological replicates. *B*, treatment of RPE cells with the indicated anti-miRs, and siRNAs does not affect cell cycle distribution. Representative cell cycle distribution was from one of two biological replicates. *C*, TSSG induced by miRNA inhibitor treatment is KDM4A-dependent. Quantification of FISH analysis. Data represent the average of two biological replicates. *Error bars* represent the S.E. * indicates significant difference from control (*CTRL*) by two-tailed Student's *t* test (*p* < 0.05). † indicates significant difference from corresponding anti-miR treated with siCTRL by two-tailed Student's *t* test (*p* < 0.05) but was not significantly different from anti-miR *CTRL/siCTRL*.

To determine whether the hsa-mir-23a/b or hsa-mir-137 anti-miRs caused TSSG through KDM4A, we co-depleted KDM4A using siRNAs with the KDM4A-targeting anti-miRs. Depletion of KDM4A by siRNA reduced KDM4A levels in the miRNA inhibitor-treated cells (Fig. 4*A*). Cell cycle distribution was unaffected (Fig. 4*B*), whereas the reduction in KDM4A levels prevented induction of TSSG by the miRNA inhibitors (Fig. 4*C*). These results demonstrate that inhibition of hsa-mir-23a/b or hsa-mir-137 promotes TSSG in a KDM4A-dependent manner.

**FIGURE 5.**
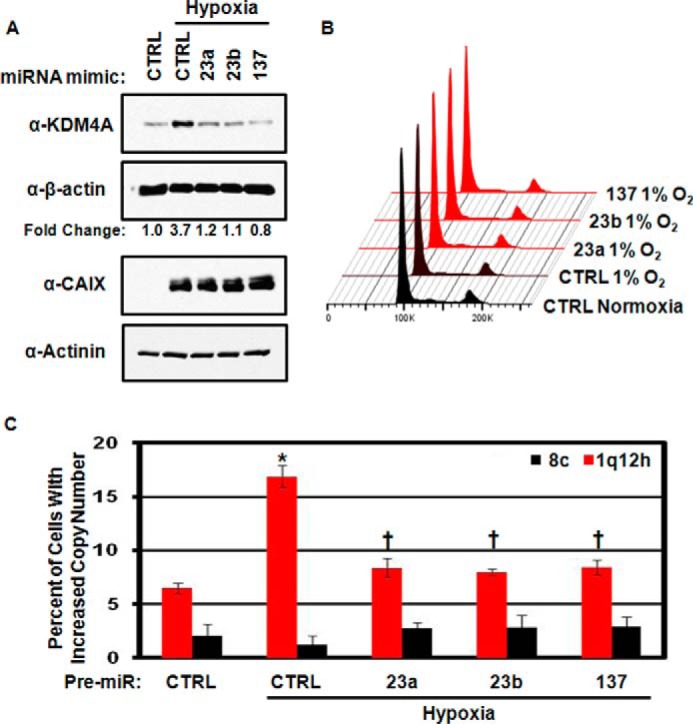
**Increased microRNA expression can ablate hypoxia-dependent TSSG.** *A*, Western blots depicting inhibition of hypoxia-dependent KDM4A induction using miRNA mimics. Representative Western blots are from one of two biological replicates. *B*, cell cycle distribution of RPE cells treated with anti-miRs. Representative cell cycle distribution was from one of two biological replicates. *C*, quantification of FISH analysis of TSSG in hypoxia-treated cells following miRNA mimic treatment. Data represent the average of two biological replicates. *Error bars* represent the S.E. * indicates significant difference from normoxia control (*CTRL*) by two-tailed Student's *t* test (*p* < 0.05). † indicates significant difference from hypoxia control by two-tailed Student's *t* test (*p* < 0.05) but was not significantly different from the normoxia control.

We previously demonstrated that hypoxia could induce TSSGs by stabilizing KDM4A protein levels (11). Because KDM4A protein levels respond to miRNAs, we reasoned that miRNA mimics would deplete KDM4A during hypoxia and prevent hypoxia-induced TSSGs. To test this hypothesis, we transfected RPE cells with miRNA mimics to hsa-mir-23a/b or hsa-mir-137 for 48 h prior to moving the cells to hypoxia for 24 h. Introduction of the miRNA mimics was sufficient to blunt the increased expression of KDM4A in hypoxia (Fig. 5*A*), but did not alter the cell cycle distribution of the treated cells (Fig. 5*B*). Consistent with the reduction in KDM4A levels, miRNA mimics were sufficient to abrogate hypoxia-dependent TSSG in RPE cells (Fig. 5*C*). Our results suggest that increasing hsa-mir-23a/b or hsa-mir-137 levels could be effective in reducing hypoxia-induced or KDM4A-dependent TSSG.

##### Loss of hsa-mir-23a Associates with Increased CKS1B Expression in Primary Breast Tumors

**FIGURE 6.**
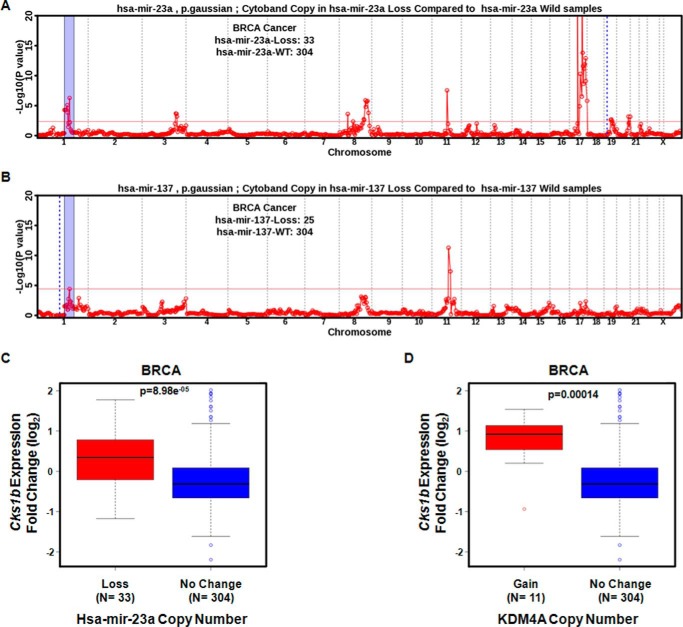
**hsa-mir-23a loss in breast cancer correlates with 1q12-21 copy gain and *CKS1B* expression.** *A*, TCGA primary breast tumor samples with loss of hsa-mir-23a have an enrichment for copy gain of 1p11.2 through 1q23.3 (*blue-shaded region*). *Dashed blue line* indicates genomic location of the indicated miRNA. *B,* TCGA primary breast tumor samples with loss of hsa-mir-137 have enrichment for copy gain of 1p11.2 through 1q23.3 (*blue shaded region*). The *blue dotted line* indicates genomic location of the indicated miRNA. *C* and *D*, expression of the drug resistance oncogene *CKS1B* is increased in tumors with loss of hsa-mir-23a (*C*) or gain of *KDM4A* (*D*). The Wilcoxon *p* value is indicated in each box-plot.

We further substantiated our *in vitro* findings by analyzing primary breast tumors (BRCA) in TCGA. Specifically, we evaluated tumors that presented a loss of each miRNA alone and did not present another miRNA loss or *KDM4A* amplification or *KDM4A* loss. Using these criteria, we observed a significant gain for the 1p11.2–1q22 region in tumors presenting a loss for hsa-mir-23a (Fig. 6*A*). Tumors with loss of the other miRNAs showed a modest significance for this region (hsa-mir-137; Fig. 6*B*) or no significance (hsa-mir-23b; data not shown). Previously, we demonstrated that breast tumors and breast cancer cells were able to generate copy gain and increased expression of the drug-resistant oncogene *CKS1B* (located at 1q21.3 inside the amplified region of tumors with hsa-mir-23a loss) upon hypoxic exposure (11). We asked whether BRCA tumors with hsa-mir-23a loss had increased *CKS1B* expression. We observed that loss of hsa-mir-23a had increased *CKS1B* that was comparable with the increase in expression observed for samples with *KDM4A* amplification (Fig. 6, *C* and *D*). Loss of hsa-mir-23b or hsa-mir-137 resulted in a comparable trend for increased *CKS1B* expression, but it was not significant (data not shown).

##### MicroRNAs Regulate Copy Number and Expression of the Drug-resistant Oncogene CKS1B

**FIGURE 7.**
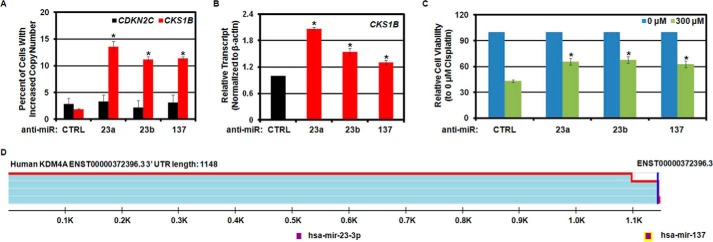
**Regulation of *CKS1B* copy number and expression by miRNAs correlates with a reduced response to cisplatin.** *A,* treatment of MDA-MB-231 cells with anti-miRs induces copy gain of *CKS1B* but not the control region *CDKN2C*. Quantification of FISH analysis is shown. Data represent the average of two biological replicates. *Error bars* represent the S.E. * indicates significant difference from control (*CTRL*) by two-tailed Student's *t* test (*p* < 0.05). *B*, treatment of MDA-MB-231 cells with anti-miRs induces expression of *CKS1B*. Data represent the average of two biological replicates. *Error bars* represent the S.E. * indicates significant difference from control by two-tailed Student's *t* test (*p* < 0.05). *C*, treatment of MDA-MB-231 cells with anti-miRs reduced the response to 300 μm cisplatin. Cells were plated and transfected with the indicated anti-miRs. 24 h later, vehicle (0.9% NaCl) or 300 μm cisplatin was added. Cell survival was measured 48 h later by MTT assay. Data represent the average of eight biological replicates measured in technical quadruplicate. *Error bars* represent the S.E. * indicates significant difference from control by two-tailed Student's *t* test (*p* < 0.05). *D,* TargetScan 7.0 UTR schematic depicting reduced read count at KDM4A 3′-UTR, which would remove hsa-mir-137 seed sequence in some transcripts. Adapted from TARGETSCAN 7.0.

Copy gain and increased expression of *CKS1B* are associated with poor patient outcome and drug-resistant cancer (4, 20–23). Understanding mechanisms that can increase *CKS1B* levels has important clinical implications. Because *CKS1B* copy gain and increased expression in hypoxia were KDM4A-dependent, we hypothesized that hsa-mir-23a/b and hsa-mir-137 would promote gain and increased expression for *CKS1B*. To directly test this hypothesis, we transfected breast cancer cells (MDA-MB-231) with miRNA inhibitors for hsa-mir-23a, hsa-mir-23b, or hsa-mir-137 and assessed copy gain and gene expression. All miRNA inhibitors resulted in increased KDM4A protein levels (Fig. 2*D*), while not altering the cell cycle profile (Fig. 2*F*). *CKS1B* was gained when cells were treated with hsa-mir-23a/b or hsa-mir-137 anti-miRs and had increased mRNA levels (Fig. 7, *A* and *B*), which was consistent with our recent findings in hypoxia (11). Because increased copy number and expression of *CKS1B* has been linked to cisplatin resistance (20, 23, 29, 30), we hypothesized that the anti-miR induction would promote resistance to cisplatin in breast cancer cells. Indeed, prior treatment with inhibitors to hsa-mir-23a/b or hsa-mir-137 resulted in decreased sensitivity to cisplatin (Fig. 7*C*). Taken together, our data demonstrate that miRNAs modulate *CKS1B* gains and expression *in vitro* and show an association *in vivo*, which provides another mechanism for increased levels of this oncogene that may contribute to resistance to cisplatin and other drugs in cancer.

## Discussion

Our results demonstrate that miRNAs can impact copy number heterogeneity through the regulation of TSSGs by directly regulating a chromatin-modifying enzyme. Our data also suggest copy number gains and expression may also be affected by alterations in how miRNAs are expressed. For example, alterations in chromatin-related enzymes, transcription factors, or environmental conditions could impact miRNA levels and in turn copy gains and gene expression. Taken together, these findings illustrate the impact that miRNAs could have on transient genome stability through chromatin modulation, which opens a new perspective on how non-coding RNAs could be involved in modulating tumor heterogeneity and potentially promoting phenotypes such as drug resistance. These data also emphasize the need to map the chromatin factors and the miRNAs that regulate them and are involved in TSSGs.

Our data raise the possibility that extrinsic and intrinsic factors could modulate the composition of miRNAs within single cells or a population of cells to control the frequency of TSSGs. Alternatively, cells could alter the 3′-UTR length of key TSSG modulators and, in turn, increase heterogeneity that may impact phenotypes such as drug resistance. It remains possible that altered 3′-UTR length could be important in regulating KDM4A protein levels within tumors because amplification, altered stability, and miRNAs are instrumental in regulating KDM4A levels (12, 15, 17, 18). Consistent with this idea, TARGETSCAN7.0 indicates that the *KDM4A* 3′-UTR can use an alternative polyadenylation site that would eliminate the hsa-mir-137 site from the 3′-UTR (Fig. 7*D*) (31). Loss of the miRNA site from the 3′-UTR could result in increased KDM4A and in turn promote TSSGs. Alternatively, cells could select for differential 3′-UTR usage, which is frequently observed in cancer cells (32, 33). Differential use of 3′-UTRs without miRNA binding sites could also increase KDM4A levels and promote TSSG and copy number heterogeneity. Future studies need to establish whether *KDM4A* transcripts have altered 3′-UTR lengths that are associated with TSSG-associated copy number heterogeneity.

MicroRNAs are often misregulated in cancer, and hsa-mir-23a/b and hsa-mir-137 are no exception (34–41). For example, reduced expression of hsa-mir-137 and hsa-mir-23b has been implicated in cisplatin resistance in solid malignancies (34, 37). Consistent with these previous observations, we observed copy gain and up-regulation of *CKS1B*, which is a cell cycle regulator that has been linked to and promotes cisplatin and other drug resistance in myeloma, breast cancer, and non-small cell lung cancer (20, 23, 29, 30). Therefore, tumors carrying the loss of hsa-mir-23a/b and hsa-mir-137 or the mis-regulation of miRNAs could mediate changes in cisplatin response by regulating KDM4A protein levels, promoting transient site-specific copy gains, and heterogeneous overexpression of *CKS1B*. For these reasons, it may be beneficial to consider using hsa-mir-23a/b and hsa-mir-137 mimics or *KDM4A* antisense RNA strategies to reduce KDM4A protein levels in tumors that have lost these miRNAs or gained *KDM4A*. In fact, miRNA mimics and inhibitors are gaining traction in their use as therapies for metabolic disease and cancer (42, 43). As new regulators of TSSGs are identified, it will be important to evaluate how they are regulated and consider miRNAs as a potential way to modulate their activity.

Our work underscores how changes in miRNA abundance could influence how tumors acquire intra-tumoral copy-number heterogeneity. The copy number changes we describe in cell culture models are transient. We observed correlations for these gains in primary tumors; however, future studies need to establish whether the copy number variations associated with miRNA loss in tumors are present in all cancer cells and whether they are transient (*i.e.* in S-phase only). Most of the miRNA loss events we observed were in the GISTIC (−1) category reflecting low level loss (as opposed, for example, to homozygous deletions) often caused by whole-chromosome or chromosome arm loss. This suggests miRNA loss events are not providing strong fitness advantage to the cells, but perhaps promote heterogeneity and plasticity that may serve as the basis of future selection. The observed intra-tumoral heterogeneity is likely the result of both permanent and transient heterogeneity. Uncovering how inappropriately amplified regions are lost will help identify pathways that may be mis-regulated in cancer leading to the accumulation and perhaps also inheritance of specific genomic regions. We hypothesize that other defects in cancer cells could then promote incorporation of the TSSGs and, in turn, potentially have a permanent contribution to drug resistance. It still remains to be determined whether TSSGs could, or need to, become permanent and spread across the entire cancer cell population to promote cellular phenotypes such as drug resistance. Therefore, future studies should directly address whether amplified regions in tumors are stable or transient in nature.

In conclusion, this study highlights the important link between chromatin modulation, miRNA levels, heterogeneous and transient site-specific copy-number gains, and potential phenotypes such as drug resistance. These findings also reiterate the importance in mapping the pathways and enzymes that are contributing to TSSGs and how the transient overexpression of genes may have a lasting effect on the cancer, even if present only transiently in a subset of the cells (such as potentiating drug resistance). Uncovering these associations will have a profound impact on our understanding of transient copy number heterogeneity and its potential effect on cancer. Ultimately, these networks may identify novel biomarkers and drug targets for cancer genome heterogeneity.

## Author Contributions

J. C. B. and J. R. W. designed the experiments, analyzed data, and prepared the manuscript. J. C. B. conducted the experiments in Figs. 1–5 and 7. H. Z., J. K., and G. G. analyzed and prepared figures related to the TCGA analysis in Fig. 6. All authors reviewed the manuscript.

## Footnotes

5 The abbreviations used are:

TSSG: transient site-specific copy gain
HU: hydroxyurea
MTT: 3-(4,5-dimethylthiazol-2-yl)-2,5-diphenyltetrazolium bromide
MT: mutant
TCGA: The Cancer Genome Atlas
miR: microRNA.
